# Genomic and Functional Analysis of Carbohydrate Esterases in the Maize Pathogen *Exserohilum rostratum*

**DOI:** 10.3390/microorganisms13112588

**Published:** 2025-11-13

**Authors:** Zi-Ming Wang, Zi-Qi Wang, Hong-Xia Yuan, Meng-Jin Liu, Cong Chen, Jian-Gang Kang, Hong-Lian Li, Ya-Fei Wang

**Affiliations:** College of Plant Protection, Henan Agricultural University, Zhengzhou 450002, China; wangzimingq8@163.com (Z.-M.W.); e2580677458@163.com (Z.-Q.W.); yhx2156@126.com (H.-X.Y.); lmj01129@163.com (M.-J.L.); thchen8901@163.com (C.C.); jiangangkang@henau.edu.cn (J.-G.K.)

**Keywords:** *Exserohilum rostratum*, carbohydrate esterases, gene family, physicochemical properties, expression patterns

## Abstract

*Exserohilum rostratum* is a causal agent of severe maize leaf spot, posing a threat to maize production. Carbohydrate esterase (CE) can catalyze the removal of acyl modifications from plant cell wall polysaccharides, thereby promoting polysaccharide hydrolysis. A total of 87 *CE* genes were identified in the *E. rostratum* ER1 genome. In this study, we conducted a comprehensive analysis of the *E. rostratum CE* (*ErCE*) genes, including physicochemical properties, structural features, promoter cis-acting regulatory elements, and functional analysis. Subcellular localization analysis revealed that more than half of ErCEs were located extracellular. ErCEs contain abundant conserved domains, indicating functional diversity of these proteins. The promoter region of *ErCE* genes contains a rich variety of cis-acting regulatory elements related to plant hormone regulation, stress response, and developmental processes. Functional enrichment analysis indicated that *ErCE* genes are predominantly involved in metabolic pathways. In addition, the expression pattern revealed significant changes in *ErCE* genes during *E. rostratum* infection, indicating that they play an important role in pathogen invasion and lesion expansion. Overall, this study elucidated the structural characteristics and expression patterns of the *CE* genes in *E. rostratum*, providing conditions for further exploration of their roles in fungal pathogenesis and laying the foundation for the improvement of sustainable agricultural systems using related genes.

## 1. Introduction

Carbohydrates are the primary energy source required for sustaining life activities and play a central role in the physiology of living organisms [1]. Based on sequence characteristics, carbohydrate-active enzymes (CAZymes) in the CAZy database are divided into six categories: glycoside hydrolases (GHs), glycosyltransferases (GTs), carbohydrate esterases (CEs), polysaccharide lyases (PLs), carbohydrate-binding modules (CBMs), and auxiliary activities (AAs). As an important member of the CAZyme family, CEs play essential roles in diverse biological processes [2]. CEs catalyze the removal of ester modifications from polysaccharides, thereby enhancing the efficiency of glycoside hydrolases in degrading complex polysaccharides [3,4,5]. CEs have significant structural diversity and can catalyze the deacetylation of various polysaccharides, including chitin and plant hemicellulose [3].

The most abundant polysaccharides in nature are cellulose and hemicelluloses, which constitute plant cell walls, and chitin, which is distributed in fungal cell walls and arthropod exoskeletons [6,7,8,9,10]. These macromolecular polysaccharides provide structural support to cell walls and form a physical defense barrier, thereby enabling organisms to resist infection by external pathogenic microorganisms [11,12]. In plant cell wall polysaccharides, CEs hydrolyze O-acetyl modifications, thereby increasing the accessibility of these polymers to enzymatic degradation [3,13]. Pathogens can breach the multilayered structural barrier of plant cell walls and achieve invasion through deacetylation and cell wall-degrading enzymes that hydrolyze polysaccharide components such as cellulose, pectin, and xylan [9].

Maize is one of the three major staple crops worldwide and plays a vital role in ensuring global food security [14,15,16,17,18,19]. *E. rostratum* can infect a wide range of crops, and the maize leaf spot it causes is destructive in major production areas [20,21]. Surprisingly, this fungus has also been reported as an opportunistic human and animal pathogen [22,23,24]. At present, effectively controlling the damage caused by *E. rostratum* remains a major challenge. Biological and genetic control strategies, owing to their safety and efficiency, offer promising options for agricultural disease management [25,26,27]. Analysis of pathogenic factors associated with *E. rostratum* can provide valuable targets for the development of safe and efficient novel control agents, while also establishing a theoretical basis for breeding disease-resistant maize varieties.

In this study, we identified all *ErCE* genes in the *E. rostratum* ER1 genome and analyzed their physicochemical properties. Phylogenetic analysis was conducted based on the ErCE protein sequences, and their structural characteristics were further studied using bioinformatics methods. To explore the potential functions of *ErCE* genes, we analyzed the cis-acting regulatory elements in the promoter regions of these genes and further analyzed their expression patterns during fungal infection. These integrated analyses provide a foundation for further elucidation of the biological functions of *ErCE* genes in pathogen–host interactions.

## 2. Materials and Methods

### 2.1. Identification and Analysis of ErCE Genes

To identify *CE* family genes in *E. rostratum* ER1, we retrieved its genome (GenBank accession number: JAHUAD000000000), with detailed annotation results available in a public repository (https://zenodo.org/deposit/5386693, accessed on 5 August 2025). CE proteins in the *E. rostratum* ER1 genome were identified using the CAZy database with an E-value < 1 × 10^−5^ [2,28]. The physicochemical properties of ErCE proteins were subsequently predicted using the ExPASy ProtParam tool (https://web.expasy.org/protparam/, accessed on 5 August 2025) [29]. To further predict the potential subcellular localization of these proteins, we used the PSORT prediction server (https://wolfpsort.hgc.jp, accessed on 5 August 2025).

### 2.2. Multiple Sequence Alignment and Phylogenetic Analysis

To analyze the evolutionary relationships of ErCE proteins, all sequences were aligned using MAFFT [30]. The resulting alignment was then imported into PhyloSuite v1.2.3 [31] for Maximum Likelihood (ML) phylogenetic tree reconstruction. The ModelFinder module was used to select the best-fit substitution model. ML reconstruction was performed using WAG + I + G4 model with 1000 bootstrap replicates to assess branch support. The phylogenetic tree was subsequently uploaded to the Evolview platform (https://evolgenius.info//evolview-v2/, accessed on 7 August 2025) for visualization and refinement [32].

### 2.3. Gene Structure and Protein Domain Analysis

The gene structures of *ErCE* genes were predicted and visualized using the Gene Structure Display Server (GSDS) [33]. This analysis provided a clear illustration of exon numbers and intron distribution features of each *ErCE* gene. To identify the motifs in ErCE proteins, their amino acid sequences were analyzed using the Multiple Em for Motif Elicitation (MEME) Suite online platform [34], with the number of motifs set to 10 in the parameter settings. The conserved domains of the protein sequences were predicted using the NCBI Conserved Domain Database (CDD) via the CD-Search tool (https://www.ncbi.nlm.nih.gov/Structure/bwrpsb/bwrpsb.cgi, accessed on 7 August 2025) with default parameters and subsequently visualized using TBtools-II v2.326 [35,36].

### 2.4. Promoter Region Analysis and Gene Ontology (Go) Annotation

The 2000 bp upstream sequences of *ErCE* genes were extracted using TBtools-II (v2.326). Cis-acting regulatory elements (CAREs) within these upstream sequences were predicted using PlantCARE [37] and subsequently visualized with TBtools-II (v2.326). To further investigate the functional characteristics of *ErCE* genes, GO enrichment analysis was performed using the OmicShare bioinformatics platform [38].

### 2.5. Analysis of ErCE Expression Patterns

Based on previous studies, we conducted the detached leaf assay for evaluating transcriptome changes during infection [21,39]. Leaves of healthy 3-week-old maize plants (B73 inbred line) were cut into fragments of approximately 3–4 cm and placed in Petri dishes lined with sterile filter paper moistened with sterile distilled water to maintain high humidity. Leaf fragments were inoculated with a conidial suspension (1 × 10^5^ conidia/μL). After 24 h of pathogen inoculation, the hyphae of the pathogen mainly invade through stomata. After 48 h, the mycelium has penetrated the host cell, causing structural damage, tissue browning, and widespread necrosis. After 72 h, dense lesions appeared on the leaves and sheaths, indicating severe disease progression (unpublished data). The samples collected at 0 h served as the spore control, while infected samples were harvested at 24, 48, and 72 h post-inoculation for further analysis. Inoculated samples were incubated in a controlled growth chamber at 25 °C under a 12 h light/12 h dark photoperiod with relative humidity ≥ 90%. Three biological replicates were included for each time point. After collection, samples were immediately frozen in liquid nitrogen and sent to Novogene Co., Ltd. (Beijing, China) for total RNA extraction and transcriptome sequencing. Differential expressions of *ErCE* genes were visualized by generating heatmaps based on FPKM values using TBtools-II (v2.326), thereby illustrating their expression patterns.

### 2.6. RT-qPCR

Total RNA was extracted from the spore and the maize leaf samples collected at 24, 48, and 72 h after spore inoculation using the RC411-01 Total RNA Extraction Kit (Vazyme, Nanjing, China). First-strand complementary DNA (cDNA) was synthesized using the HiScript IV RT SuperMix for qPCR (+gDNA wiper) (Vazyme, Nanjing, China), according to the manufacturer’s instructions. Primers were designed using the Primer3Plus online tool (https://primer3plus.com, accessed on 10 August 2025), with product lengths set to 150–250 bp and other parameters at default values. Primer specificity and potential dimer formation were assessed using TBtools-II (v2.326). The final primer sequences were provided in Supplementary Table S4. Quantitative PCR (qPCR) was performed using ChamQ Blue Universal SYBR qPCR Master Mix (Vazyme, Nanjing, China) in a 20 μL reaction system containing 10 μL of 2× SYBR Green Master Mix, 0.4 μL each of forward and reverse primers (10 μM), 2 μL of diluted cDNA template, and 7.2 μL of RNase-free water. The amplification program consisted of an initial denaturation at 95 °C for 30 s, followed by 40 cycles of 95 °C for 10 s and 60 °C for 30 s (annealing/extension). Three biological replicates were conducted for each sample, with three technical replicates per biological replicate. Relative expression levels were calculated using the 2^−ΔΔCt^ method [40], and the results were visualized with GraphPad Prism 10.5.

## 3. Results

### 3.1. Identification and Physicochemical Property Analysis of Carbohydrate Esterase Genes

A total of 87 *ErCE* genes were identified in the *E. rostratum* ER1 genome (designated *ErCE1*–*ErCE87*), and their physicochemical properties were evaluated. The detailed characteristics were provided in Table 1. ErCE proteins belong to 12 different superfamilies, with 26 ErCE proteins belonging to CE10, 22 belonging to CE1, and 15 belonging to CE5. The molecular weights of these proteins ranged from 20.48 kDa (ErCE16) to 155.35 kDa (ErCE49). The length of these proteins ranged from 206 to 1399 amino acid. The predicted isoelectric points (pI) of these proteins ranged from 4.95 (ErCE31) to 9.51 (ErCE64), comprising 46 acidic proteins (pI < 6.5), 32 basic proteins (pI > 7.5), and 9 neutral proteins (6.5 < pI < 7.5). The grand average of hydropathicity (GRAVY) values ranged from −0.63 (ErCE63) to 0.32 (ErCE16), with 72 proteins predicted to be hydrophilic (GRAVY ≤ 0) and 15 predicted to be hydrophobic (GRAVY > 0). Subcellular localization analysis revealed that ErCE proteins were predominantly distributed in the extracellular space (45), cytoplasm (19), and mitochondria (15). Collectively, these results indicate that the CE proteins in *E. rostratum* exhibit complex composition and diverse physicochemical properties, suggesting their potential roles in fungal growth, development, and pathogenicity.

### 3.2. Phylogenetic Analysis

**Figure 1:**
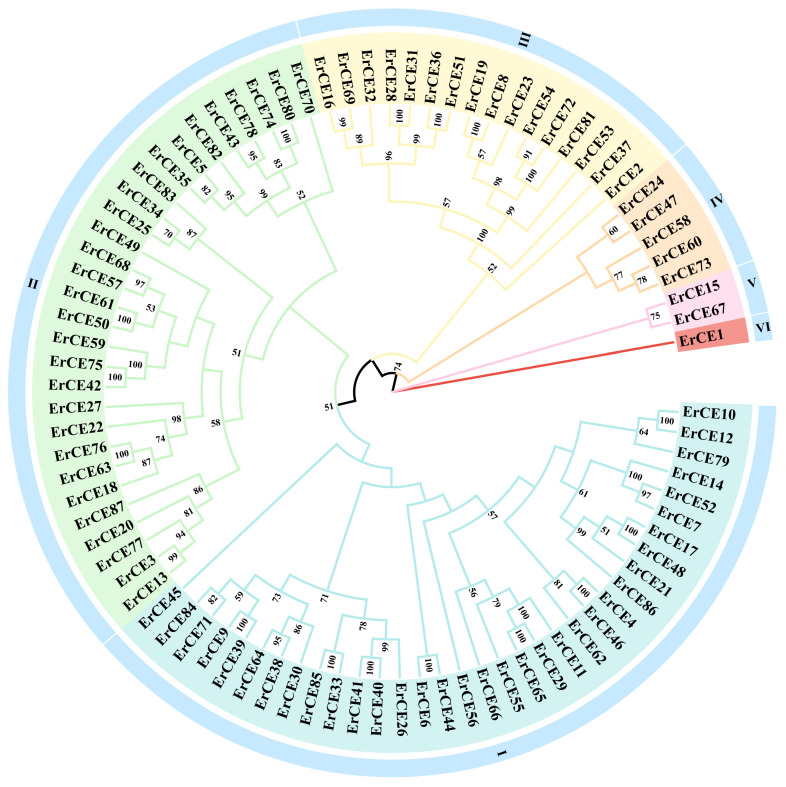
Maximum likelihood phylogenetic tree based on ErCE protein sequences. “Er” denotes *E. rostratum*. The bootstrap values below 50% have been cut off. Based on the phylogenetic tree, all ErCE proteins were classified into six groups, designated as Group I to Group VI.

To investigate the evolutionary relationships among 87 ErCE proteins, a phylogenetic tree was constructed using the ML method in PhyloSuite v1.2.3. As shown in Figure 1, the ErCE proteins were divided into six groups, with most members distributed in Group I and Group II. Group I contained the largest number of members (34), followed by Group II (29), while Group VI had the fewest members (1). The distribution of ErCE proteins among different groups reflects the sequence diversity within the CE family. ErCE sequences located in the same group have closer evolutionary relationships, while ErCE sequences located in different groups have farther evolutionary relationships.

### 3.3. Structural Analysis of ErCEs

**Figure 2:**
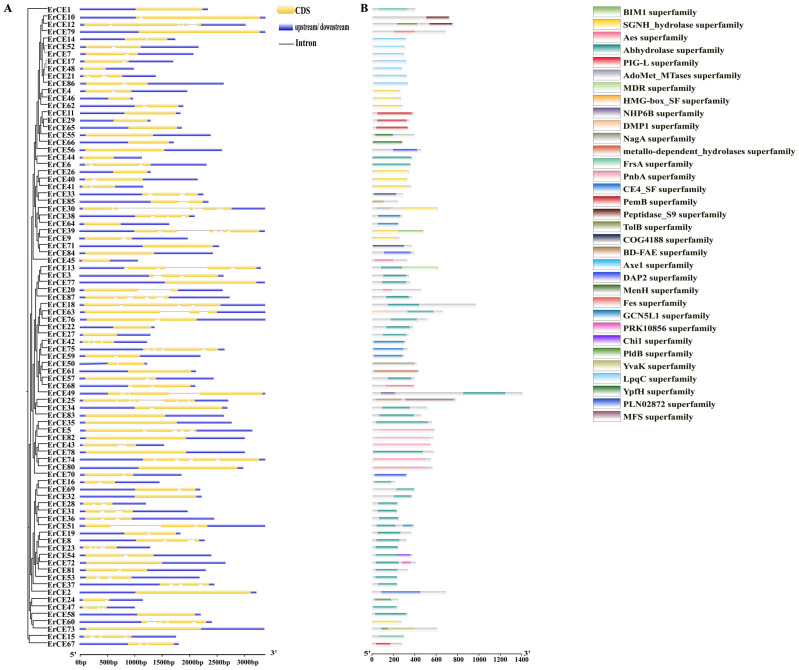
Gene structure and domain analysis of *ErCEs*. (**A**) Exon–intron structures of *ErCEs*. Blue boxes represent untranslated regions (UTRs); yellow boxes represent exons; black lines represent introns. (**B**) Domain organization of ErCE proteins. Colored rectangles indicate superfamily domains of ErCEs.

The results of gene structural analysis showed that *ErCE30* and *ErCE25* each contained six introns, whereas 30 *ErCE* genes lacked introns (Figure 2A). Genes lacking introns may exhibit higher transcriptional efficiency [41], while genes containing a greater number of introns may play important roles in splicing regulation, thereby increasing protein diversity and complexity [42]. This variation among *ErCE* genes suggests potential intron loss and gain events during the evolutionary trajectory of the family, which may contribute to functional divergence. To further analyze the characteristics of ErCE proteins, the domain analyses were conducted. ErCE proteins were found to contain a wide range of conserved superfamily domains, mainly including the Abhydrolase superfamily (35), SGNH_hydrolase superfamily (10), LpqC superfamily (7), and others (Figure 2B). Notably, the Abhydrolase superfamily was widely distributed among the family members, suggesting its potential importance in plant cell wall degradation.

**Figure 3:**
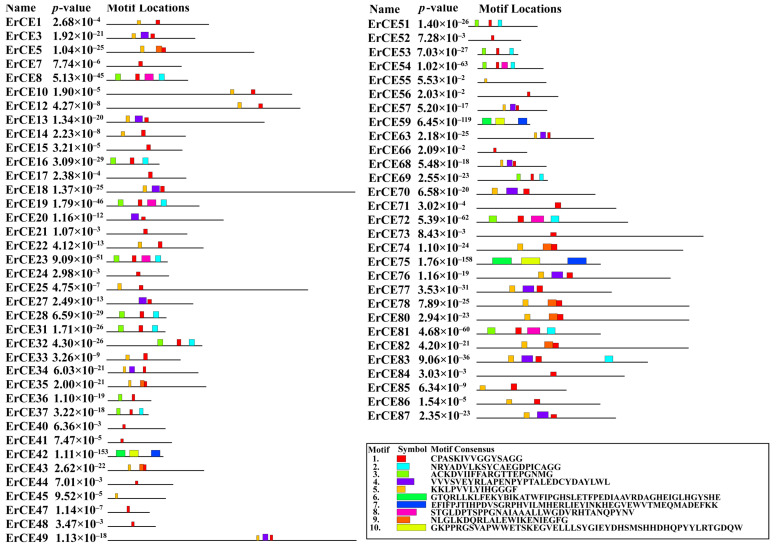
Motif prediction of ErCEs using MEME. Boxes in different colors represent distinct motifs that vary in size and sequence.

To further investigate conserved motifs of ErCE proteins, the analysis was carried out using the MEME tool, and 10 distinct motifs were identified (Figure 3 and Figure S1; Table S1). The results revealed that the number and arrangement of motifs in the ErCE proteins were different. Sixty-two ErCE proteins contained motif 1 and thirty-one contained motif 5, indicating that these two motifs are highly conserved within the family and may constitute core functional regions of the proteins. Proteins sharing similar motif compositions generally exhibited closer evolutionary relationships and may possess similar functional characteristics.

### 3.4. Analysis of Promoter Regulatory Elements of ErCEs

**Figure 4:**
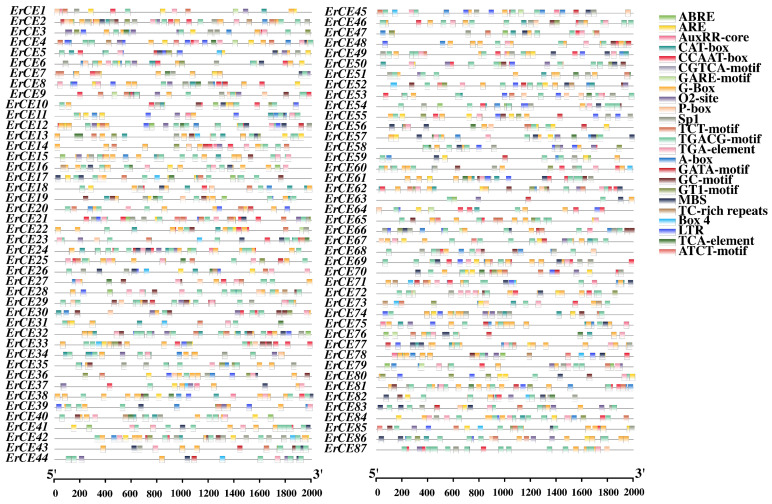
Cis-acting regulatory element (CARE) analysis in promoter regions of *ErCE* genes. Colored boxes represent different CAREs and their corresponding positions.

CARE analysis of the 2000 bp upstream promoter regions of *ErCE* genes was performed using PlantCARE. The results revealed that the promoters of *ErCE* genes harbor numerous CAREs associated with plant hormonal regulation, light responsiveness, defense, and stress responses (Figure 4 and Figure S2; Table S2). Light-responsive elements included G-box (399), Sp1 (97), TCT-motif (68), GT1-motif (54), GATA-motif (31), Box 4 (24), and ATCT-motif (10), suggesting that light signaling may be associated with *ErCE* genes. Plant hormone-related CAREs were also abundant, particularly ABRE (335) related to abscisic acid (ABA), as well as CGTCA-motif (346) and TGACG-motif (346) related to methyl jasmonate (MeJA), suggesting these *ErCE* genes may be involved in ABA- and MeJA-mediated signaling pathways. Other plant hormone-responsive elements included the auxin-responsive TGA-element (100) and AuxRR-core (16), the gibberellin-responsive P-box (42) and GARE-motif (19), and the salicylic acid–responsive TCA-element (32), implying that *ErCE* genes may participate in multiple plant hormone signaling networks. Several stress-related CAREs were also identified, including ARE (107) and GC-motif (61) associated with anaerobic induction, LTR (67) related to low-temperature responsiveness, MBS (81) involved in drought inducibility, and TC-rich repeats (18) connected to broad-spectrum stress and defense responses. These findings suggest that *ErCE* genes may play critical regulatory roles in mediating the responses of *E. rostratum* to diverse environmental stresses. In addition, development- and metabolism-related CAREs were detected, including CAT-box (78) related to meristem expression, CCAAT-box (105) serving as a binding site for MYB transcription factors, and O2-site (71) associated with zein metabolism regulation, suggesting that these *ErCE* genes may function in specific tissues or developmental stages.

### 3.5. GO Enrichment Analysis of ErCE Genes

**Figure 5:**
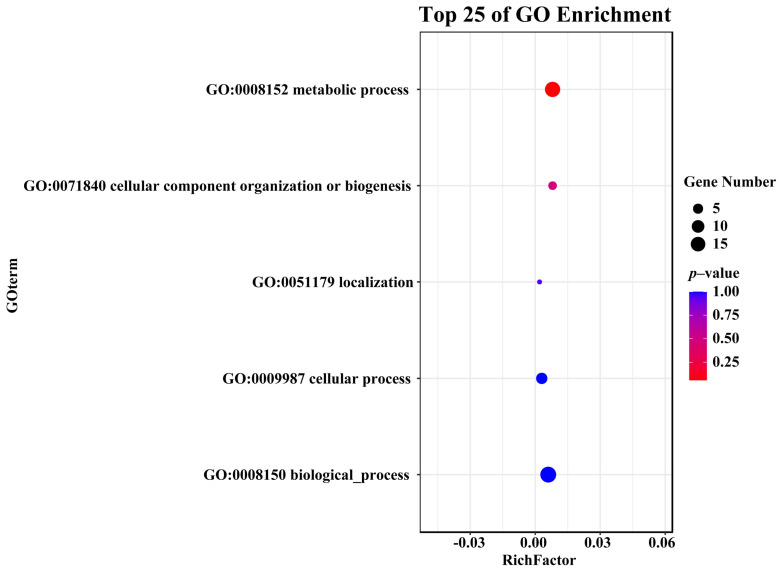
Gene ontology enrichment analysis of *ErCE* genes.

GO enrichment analysis revealed that *ErCE* genes were primarily enriched in metabolic process (GO:0008152), cellular component organization or biogenesis (GO:0071840), localization (GO:0051179), cellular process (GO:0009987), and biological process (GO:0008150) (Figure 5; Table S3). Among these, the most genes were significantly enriched in metabolic process (GO:0008152), suggesting that the *ErCE* genes mainly take part in metabolic regulation.

### 3.6. Expression Pattern Analysis of ErCE Genes

**Figure 6:**
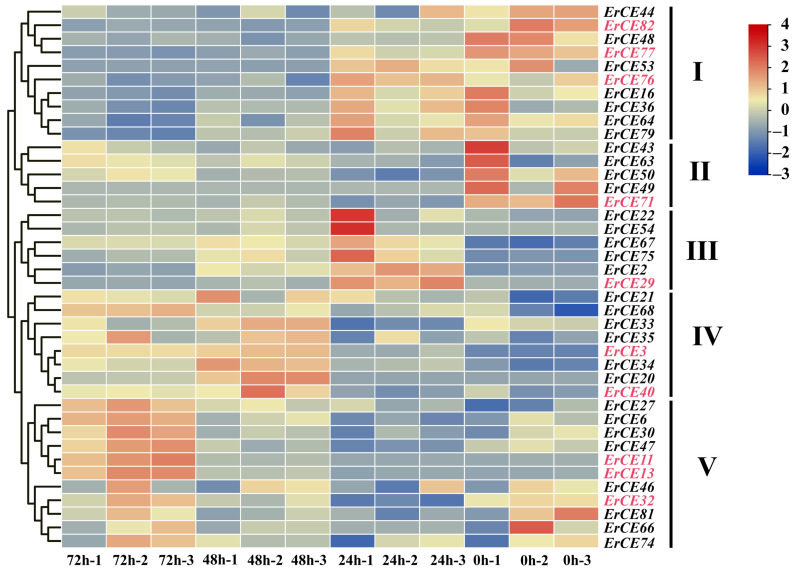
Expression pattern of *ErCE* genes based on RNA-seq data. Red and blue indicate high and low expression levels of *ErCEs*, respectively. Genes with no detectable expression are not shown. These *ErCE* genes were grouped into five clusters (I–V) based on the expression pattern.

**Figure 7:**
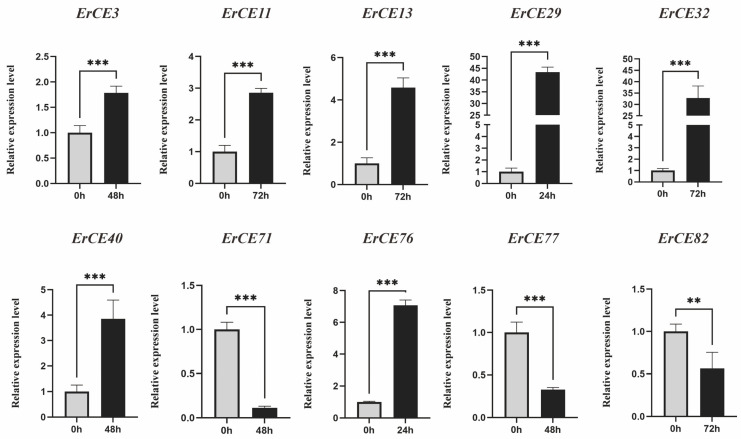
RT-qPCR validation of *ErCE* expressions. Bar charts represent the mean ± standard error (SE) of three technical replicates (** *p* < 0.01,*** *p* < 0.001).

We analyzed the expression pattern of *ErCE* genes at different times (0, 24, 48 and 72 h) during the infection process. *ErCE* genes were grouped into five clusters (Clusters I–V) based on their expression changes (Figure 6). Genes in Cluster I exhibited high expression at 0 and 24 h, suggesting their potential roles mainly focus on fungal growth, development, and early adhesion. Genes in Cluster II showed high expression at 0 h, indicating the function of these genes is primarily associated with fungal growth and development. Genes in Cluster III were significantly upregulated at 24 h, implying these genes were involved in the early stages of host infection and pathogen colonization. Genes in Cluster IV displayed pronounced upregulation at 48 h, suggesting their function was associated with pathogen expansion in host tissues during the biotrophic stage. Genes in Cluster V were highly expressed at 72 h, indicating their potential function was involved in late-stage lesion development. To validate the transcriptome data, ten *ErCE* genes were randomly selected, and specific primers were designed for RT-qPCR (Table S4). *GAPDH* was used as the internal reference gene to normalize the RT-qPCR data. The expression patterns obtained from RT-qPCR were consistent with the transcriptome data, confirming the reliability of RNA-seq data (Figure 7). Collectively, the differential expression of *ErCE* genes at distinct time points suggests that their functions have shown a clear differentiation, including host penetration, maintenance of essential physiological activities, and late-stage lesion development.

## 4. Discussion

CEs play an important role in the degradation of plant cell walls and promote the invasion of pathogens into plant tissues [13]. Therefore, the systematic identification and functional analysis of *CE* genes in *E. rostratum* can not only provide insights into the mechanism of plant cell wall disruption mediated by CEs but also establish a foundation for identifying potential targets for disease management.

In this study, a total of 87 *ErCEs* were identified in this study. Compared with another corn pathogenic fungus, *Colletotrichum graminicola*, the total number of *CE* genes in *E. rostratum* is significantly reduced. According to a previous report, there are 128 *CE* genes in *C. graminicola* [28], and the difference in the number of *CE* genes between the two corn pathogenic fungi may be closely related to their different taxonomic positions. All ErCEs were classified into different superfamilies. Among them, CE1, CE5, and CE10 had the most members, which were capable of degrading diverse substrates and thereby contributing to plant cell wall degradation and pathogen invasion [43,44]. Most ErCE proteins exhibited hydrophilic and acidic properties. Subcellular localization analysis indicated that these proteins were localized at different positions, suggesting functional diversification across different cellular compartments. Specifically, 45 ErCE proteins were predicted to be secreted into the extracellular space, where they are likely involved in plant cell wall degradation and infection. This observation is consistent with the known functions of CEs, which primarily act on cell walls and play critical roles in plant tissue invasion [13]. In addition, 15 ErCE proteins were predicted to localize to mitochondria. As essential organelles in eukaryotic cells, mitochondria are responsible for ATP production and participate in multiple key physiological processes [45]. Previous studies have demonstrated that fungal energy demand increases significantly during infection, and that mitochondrial function is crucial for fungal pathogenicity [46,47,48]. Therefore, these mitochondria-localized ErCE proteins may contribute to metabolic regulation and enhance the capability of infection and environmental adaptability by modulating energy supply.

The number of introns varied substantially among *ErCE* genes. For example, *ErCE30* and *ErCE25* contained six introns, whereas 30 *ErCE* genes lacked introns. Genes with fewer introns mean more compact structures that are generally associated with higher transcriptional efficiency [41,49], whereas *ErCE* genes with a larger number of introns may play important roles in the regulation of alternative splicing [42]. All differentially expressed *ErCE* genes were assigned to 10 different subfamilies, exhibiting remarkable structural and functional diversity. The subfamily diversity indicates that *ErCEs* may cooperate through diverse metabolic pathways or substrate recognition mechanisms to facilitate infection and host adaptation. In addition, six differentially expressed genes encode proteins with two different structural domains, indicating that these genes may have multifunctional properties and may play a broader role in the interaction between pathogens and plants. This structural diversity likely reflects functional divergence within the *ErCE* genes, and this is worthy of in-depth research.

Because protein motifs are closely associated with biological functions, they are likely to participate in processes such as pathogenicity, cell wall degradation, and immunity [50]. Motif analysis of ErCE proteins revealed that proteins in the same group generally shared similar motifs, suggesting that they may perform similar functions. GO enrichment analysis showed that 17 *ErCE* genes were enriched in metabolic processes. Eleven protein sequences encoded by these genes contained motif 1, indicating that motif 1 may be related to metabolic function. It is worth noting that among the 40 differentially expressed gene-encoded protein sequences, 29 contain motif 1, indicating that this motif may be closely related to the pathogenicity of fungi. In addition, ErCE proteins were found to possess diverse domains, with the Abhydrolase superfamily being the most prevalent. Previous studies have shown that the Abhydrolase superfamily was closely related to pathogenicity, and its knockout results in a significant reduction in virulence [51]. The widespread distribution of the Abhydrolase superfamily among ErCE proteins suggests that this domain may contribute to plant cell wall degradation and pathogen infection [9]. Nearly half of the differentially expressed genes encode protein sequences containing the Abhydrolase superfamily domain, indicating their potential involvement in host cell wall degradation and adaptation to host defense mechanisms.

CAREs in promoter regions generally exert a significant influence on gene function [52,53]. Numerous studies have demonstrated that CAREs play critical roles in regulating gene expression [54,55,56]. For instance, deletion of light-responsive elements has been shown to reduce fungal pathogenicity [57], suggesting that these elements may influence virulence by regulating gene expression. In this study, a large number of light-responsive elements were identified, revealing that photoperiod may contribute to the regulation of *ErCE* expressions. Fungi can also secrete proteins that target plant hormones to facilitate infection [58]. Therefore, the abundance of plant hormone-related elements identified in this study may help fungi influence host defense mechanisms. In addition, CAREs associated with hypoxia, drought, defense, and low-temperature responses were detected. Collectively, the identified CAREs provide valuable insights into the mechanisms of how pathogens regulate growth and development, respond to stress, and adapt to external environments.

Based on subcellular localization analysis, six genes enriched in metabolic processes are located in the cytoplasm, indicating that they may mainly participate in intracellular metabolic activities, while seven genes are extracellular, indicating that they may act as secretory enzymes on the host plant cell wall. Moreover, gene function is often correlated with expression levels [59,60,61]. For example, maize pathogen *Cochliobolus heterostrophus*, genes associated with asexual development and virulence were identified through infection-specific transcriptional patterns [39]. In this study, we tested the expression profiles of *ErCE* genes throughout the infection process. Some *ErCE* genes were highly expressed at 24 h, whereas others were upregulated at 72 h, implying different expression patterns involved in the early stage of infection and lesion expansion. The differential expression of *ErCE* genes across infection stages further revealed the functional diversity of the gene family. Among the proteins encoded by differentially expressed genes, 19 proteins were located in the extracellular space, 9 proteins were located in the cytoplasm, and 8 proteins were located in the mitochondria. They might play different roles in the process of pathogen invasion of the host. For example, extracellular localized ErCE32 might be secreted to participate in pathogen invasion, while mitochondrial ErCE76 might be involved in energy metabolism or stress response. In addition, ErCE13 contained domains belonging to the Abhydrolase superfamily and MDR (multidrug resistance) superfamily, located in the cytoplasm, indicating that it might be primarily involved in metabolic regulation, intracellular signal transduction, and protein processing.

In recent years, the interactions between maize and major pathogenic fungi have attracted considerable attention. The systematic study of the *ErCE* genes not only deepens our understanding of the pathogenic mechanism of *E. rostratum* but also provides conditions for the development of targeted biopesticides and the improvement of sustainable agricultural systems using related genes. Through bioinformatics analysis, we characterized the composition and physicochemical properties of the *ErCE* genes and performed a preliminary functional assessment. However, the current conclusions are primarily derived from bioinformatics predictions and lack direct experimental validation. Future studies should focus on identifying key pathogenicity-related genes and verifying their functions through gene knockout or overexpression. In addition, elucidating the relationship between the *ErCE* genes and maize immune responses will be critical for uncovering the regulatory networks underlying the infection process.

## 5. Conclusions

In this study, we conducted a comprehensive analysis of the *ErCE* genes during *E. rostratum* infection in maize. A total of 87 *ErCE* genes were identified, and their physicochemical properties as well as subcellular localization were predicted. The various structural domains included in ErCE protein sequences indicated that their functions might be diverse. The promoter regions of the *ErCE* genes contain abundant CAREs, which are often associated with growth and development, pathogenicity, and biotic and abiotic stress. GO enrichment analysis indicated that *ErCE* genes play key roles in metabolic processes. Transcriptome analysis demonstrated that *ErCE* genes exhibited distinct expression patterns across different infection stages, implying they were involved in multiple phases of the infection process. Collectively, this study not only deepens our understanding of the *ErCE* genes but also provides conditions for the development of biopesticides and the improvement of sustainable agricultural systems.

## Figures and Tables

**Table 1:** Predicted physicochemical characteristics and subcellular localization of ErCE proteins in *E. rostratum.*

Proposed Gene Name	Gene ID	Superfamily	CDS Length (bp)	Protein Length (aa)	Mw (KDa)	pI	GRAVY	Predicted Subcellular Localization
*ErCE1*	gene00052	CE10	1197	398	44.23	7.31	0.01	plasma membrane
*ErCE2*	gene00100	CE1	2067	688	76.11	9.37	−0.30	plasma membrane
*ErCE3*	gene00197	CE10	1038	345	38.26	5.50	−0.33	cytosolskeleton
*ErCE4*	gene00211	CE12	765	254	27.41	8.62	−0.32	extracellular
*ErCE5*	gene00511	CE10	1725	574	64.41	6.05	−0.40	cytosolskeleton
*ErCE6*	gene00722	CE1	1071	356	39.54	5.69	−0.35	mitochondrion
*ErCE7*	gene00901	CE1	879	292	31.01	8.68	−0.20	extracellular
*ErCE8*	gene01208	CE5	954	317	34.57	6.31	−0.29	extracellular
*ErCE9*	gene01233	CE3	765	254	27.02	9.17	0.01	extracellular
*ErCE10*	gene01648	CE10	2166	721	79.59	4.99	−0.37	extracellular
*ErCE11*	gene01724	CE8	1155	384	42.31	8.01	−0.32	extracellular
*ErCE12*	gene01752	CE10	2262	753	83.23	5.05	−0.34	extracellular
*ErCE13*	gene01791	CE10	1845	614	66.73	6.44	−0.14	cytosol
*ErCE14*	gene01953	CE1	927	308	33.47	8.20	−0.23	extracellular
*ErCE15*	gene02162	CE7	888	295	33.13	8.36	−0.10	extracellular
*ErCE16*	gene02348	CE5	621	206	20.48	7.66	0.32	cytosol
*ErCE17*	gene02387	CE1	933	310	32.38	8.94	−0.19	extracellular
*ErCE18*	gene02405	CE10	2901	966	105.54	6.94	−0.59	mitochondrion
*ErCE19*	gene02443	CE5	1086	361	37.34	5.57	−0.17	extracellular
*ErCE20*	gene02507	CE10	1368	455	50.48	6.12	−0.18	extracellular
*ErCE21*	gene02529	CE1	945	314	34.06	7.62	−0.22	extracellular
*ErCE22*	gene02630	CE10	1134	377	41.56	8.62	−0.07	extracellular
*ErCE23*	gene02697	CE5	717	238	24.85	8.08	−0.08	extracellular
*ErCE24*	gene02783	CE1	732	243	26.12	5.21	−0.04	cytosol
*ErCE25*	gene02822	CE10	2352	783	85.80	6.07	0.26	plasma membrane
*ErCE26*	gene02976	CE16	1026	341	38.37	5.36	−0.31	extracellular
*ErCE27*	gene03003	CE10	1014	337	37.03	7.18	−0.35	cytosol
*ErCE28*	gene03150	CE5	702	233	23.71	9.06	0.16	extracellular
*ErCE29*	gene03193	CE8	1011	336	36.19	9.04	−0.23	extracellular
*ErCE30*	gene03285	CE16	1830	609	68.84	5.19	−0.43	cytosol
*ErCE31*	gene03372	CE5	690	229	24.05	4.95	0.09	extracellular
*ErCE32*	gene03377	CE5	1119	372	34.81	8.90	0.11	extracellular
*ErCE33*	gene03537	CE1	867	288	30.35	8.64	−0.23	extracellular
*ErCE34*	gene03792	CE10	1533	510	57.07	7.01	−0.49	mitochondrion
*ErCE35*	gene03826	CE10	1665	554	61.35	5.24	−0.39	extracellular
*ErCE36*	gene04203	CE5	738	245	26.11	6.16	0.08	extracellular
*ErCE37*	gene04204	CE5	693	230	24.54	8.18	0.09	extracellular
*ErCE38*	gene04569	CE4	843	280	30.13	9.13	−0.15	extracellular
*ErCE39*	gene04584	CE3	1446	481	53.40	5.59	−0.31	extracellular
*ErCE40*	gene04761	CE16	978	325	35.88	8.82	−0.27	extracellular
*ErCE41*	gene04762	CE16	1086	361	39.22	4.20	−0.35	extracellular
*ErCE42*	gene04782	CE4	942	313	35.82	5.60	−0.52	cytosol
*ErCE43*	gene05167	CE10	1629	542	60.74	6.85	−0.41	cytosol
*ErCE44*	gene05188	CE1	1107	368	40.38	5.98	−0.06	extracellular
*ErCE45*	gene05239	CE10	984	327	36.05	5.34	−0.36	cytosol
*ErCE46*	gene05385	CE12	783	260	27.97	6.52	−0.02	extracellular
*ErCE47*	gene05859	CE1	711	236	26.13	5.97	−0.31	mitochondrion
*ErCE48*	gene05861	CE1	813	270	29.52	8.99	−0.22	extracellular
*ErCE49*	gene05866	CE10	4200	1399	155.35	9.34	−0.50	mitochondrion
*ErCE50*	gene05910	CE9	1242	413	44.05	5.56	−0.16	cytosol
*ErCE51*	gene05917	CE5	1173	390	41.37	8.70	0.31	mitochondrion
*ErCE52*	gene05929	CE1	897	298	32.14	9.20	−0.17	extracellular
*ErCE53*	gene05956	CE5	690	229	23.36	8.01	−0.02	extracellular
*ErCE54*	gene06354	CE5	1116	371	36.95	5.69	0.10	extracellular
*ErCE55*	gene06398	CE1	1164	387	43.52	6.05	−0.27	peroxisome
*ErCE56*	gene06400	CE1	1365	454	51.41	5.95	−0.33	peroxisome
*ErCE57*	gene06456	CE10	1179	392	44.68	8.75	0.05	extracellular
*ErCE58*	gene06479	CE1	999	332	38.01	5.52	−0.45	mitochondrion
*ErCE59*	gene06713	CE4	891	296	33.63	6.15	−0.39	nucleus
*ErCE60*	gene06800	CE12	813	270	30.04	5.36	−0.19	cytosol
*ErCE61*	gene06868	CE9	1305	434	45.88	5.36	0.02	cytosol
*ErCE62*	gene07048	CE12	834	277	28.88	9.21	−0.11	extracellular
*ErCE63*	gene07146	CE10	1968	655	72.87	4.94	−0.63	nucleus
*ErCE64*	gene07337	CE4	753	250	28.19	9.51	−0.15	plasma membrane
*ErCE65*	gene07427	CE8	1002	333	37.06	8.81	−0.44	extracellular
*ErCE66*	gene07614	CE1	840	279	30.51	5.96	0.01	mitochondrion
*ErCE67*	gene07904	CE14	840	279	31.23	9.41	0.00	mitochondrion
*ErCE68*	gene07941	CE10	1167	388	42.10	6.97	−0.17	mitochondrion
*ErCE69*	gene08303	CE5	1188	395	41.70	6.38	−0.07	extracellular
*ErCE70*	gene08331	CE10	942	313	34.80	5.27	−0.20	mitochondrion
*ErCE71*	gene08347	CE7	1107	368	39.72	5.11	0.08	extracellular
*ErCE72*	gene08673	CE5	1200	399	41.44	4.96	−0.20	extracellular
*ErCE73*	gene08701	CE1	1821	606	65.90	5.78	−0.11	cytosol
*ErCE74*	gene09040	CE10	1635	544	59.22	5.47	−0.19	cytosol
*ErCE75*	gene09060	CE4	984	327	37.67	5.38	−0.60	cytosol
*ErCE76*	gene09462	CE10	1536	511	56.98	7.17	−0.40	mitochondrion
*ErCE77*	gene09655	CE10	1071	356	39.10	6.01	−0.19	cytosol
*ErCE78*	gene09870	CE10	1710	569	63.47	5.93	−0.28	mitochondrion
*ErCE79*	gene09871	CE1	2058	685	75.60	6.07	−0.37	extracellular
*ErCE80*	gene10109	CE1	1683	560	62.87	6.28	−0.49	mitochondrion
*ErCE81*	gene10227	CE5	984	327	34.00	5.94	−0.02	extracellular
*ErCE82*	gene10334	CE1	1704	567	61.96	7.03	−0.30	extracellular
*ErCE83*	gene10579	CE10	1356	451	50.72	7.66	−0.38	mitochondrion
*ErCE84*	gene10840	CE15	1173	390	40.94	8.65	−0.16	extracellular
*ErCE85*	gene10927	CE1	714	237	25.51	8.42	−0.28	cytosol
*ErCE86*	gene10964	CE1	981	326	35.77	5.77	−0.46	extracellular
*ErCE87*	gene10979	CE10	1104	367	40.71	5.89	−0.25	cytosol

ID: identity; bp: base pair; aa: amino acids; pI: isoelectric point; Mw: molecular weight; GRAVY: grand average of hydropathicity; KDa: kilodalton.

## Data Availability

The original contributions presented in this study are included in the article/Supplementary Materials. Further inquiries can be directed to the corresponding authors.

## Supplementary Materials

The following supporting information can be downloaded at: https://www.mdpi.com/article/10.3390/microorganisms13112588/s1, Table S1. ErCE genomic, CDS, protein and promoter sequences. Table S2. Cis-acting regulatory elements are present in the *ErCE* gene promoter regions. Table S3. Gene ontology enrichment analysis of *ErCE* genes. Table S4. The primers used in this study. Figure S1. Sequence logo conserved motif of ErCE proteins. Figure S2. The numbers of predicted CAREs in the *ErCE* promoter regions.
