# Supplementary material for: Genomic and Functional Analysis of Carbohydrate Esterases in the Maize Pathogen Exserohilum rostratum
Source: Microorganisms. 2025 Nov 13;13(11):2588. doi: 10.3390/microorganisms13112588 (PMC12654809; doi:10.3390/microorganisms13112588)
Supplement: Supplementary file 1 [file microorganisms-13-02588-s001.zip › Figure S1. Sequence logo conserved motif of ErCE proteins. Figure S2. The numbers of predicted CAR.pptx]

## Slide 1
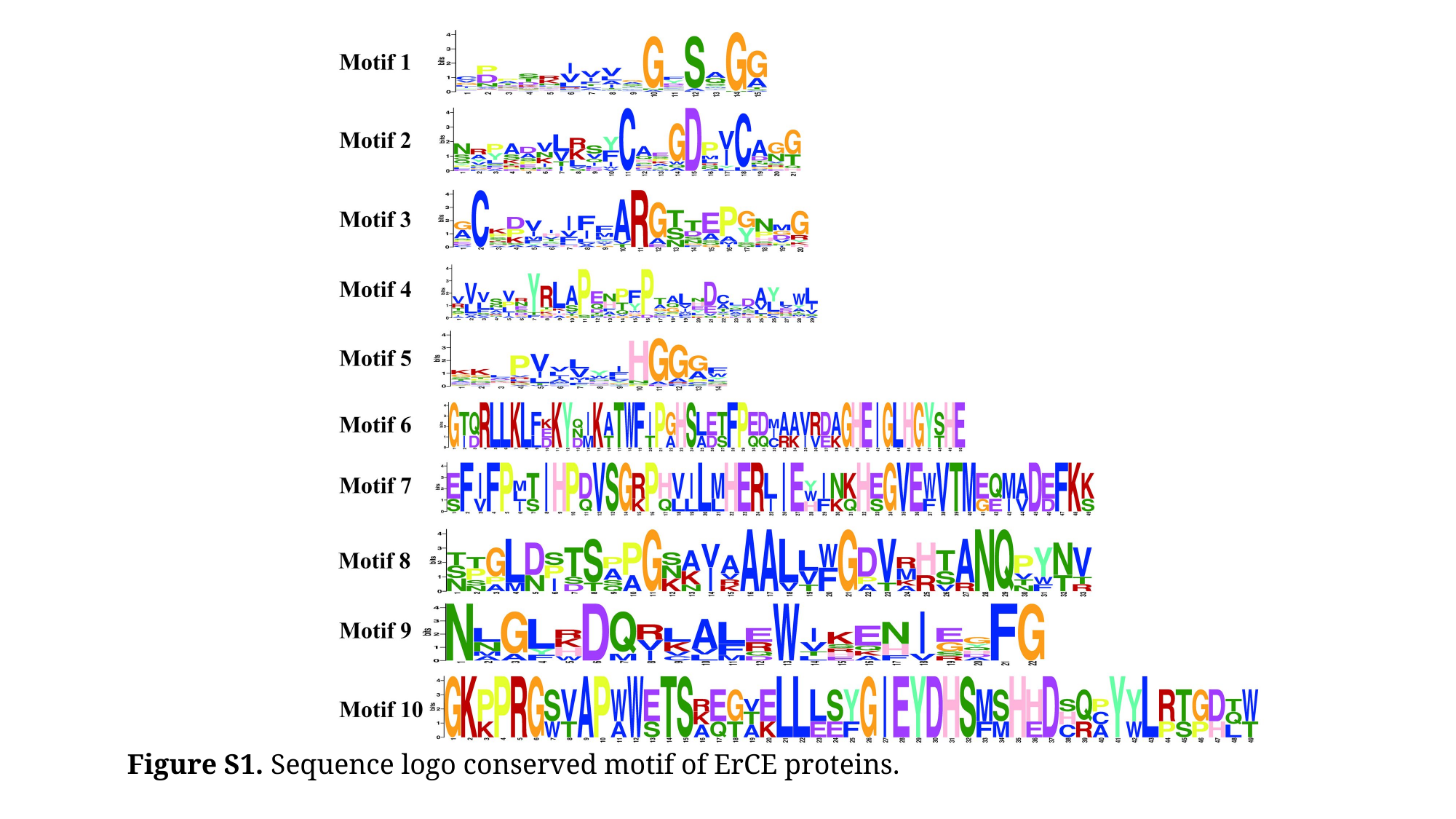

Figure S1. Sequence logo conserved motif of ErCE proteins.

## Slide 2
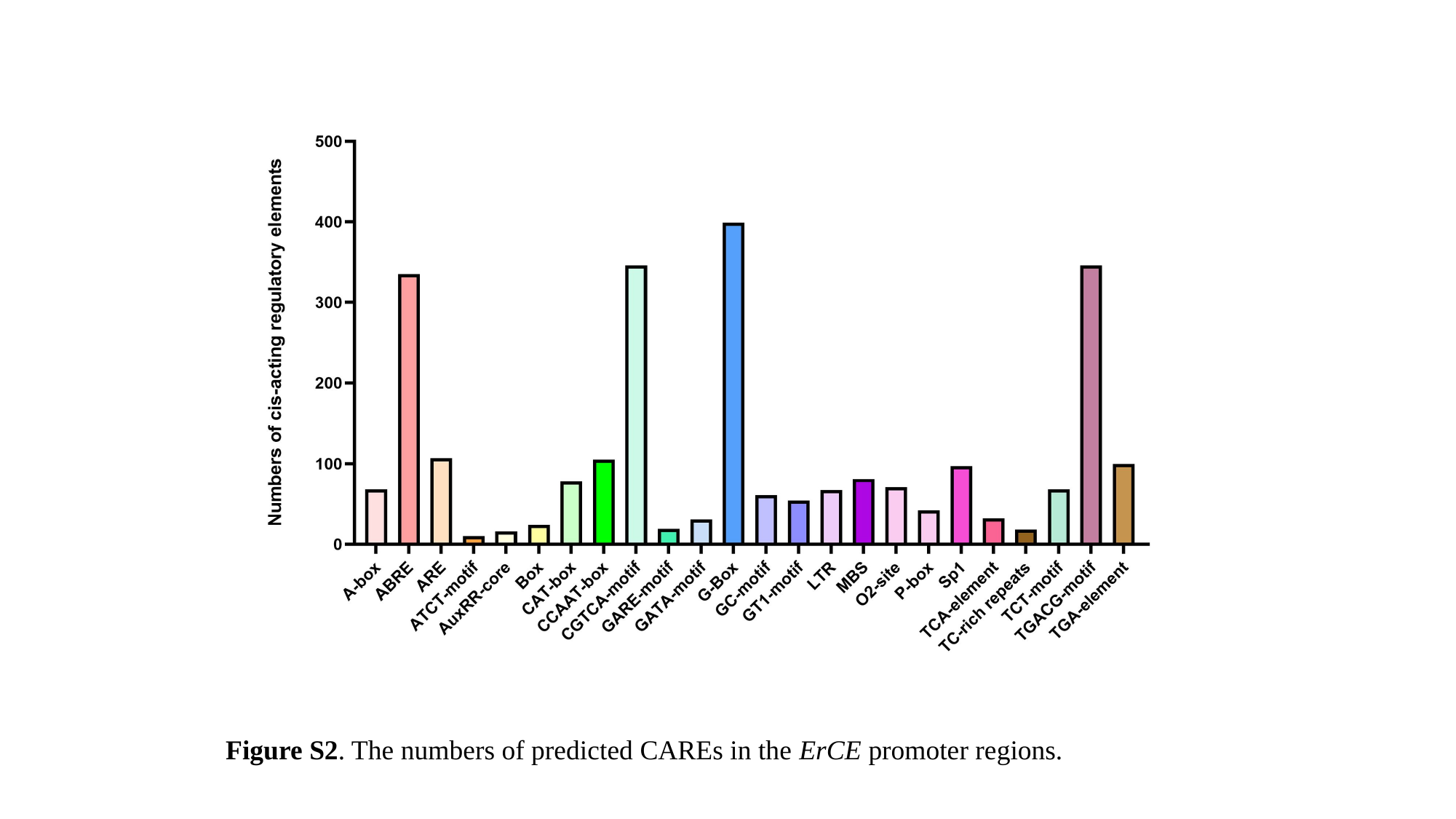

Figure S2. The numbers of predicted CAREs in the ErCE promoter regions.
